# 

**Published:** 1907-12

**Authors:** 


### BOOKS RECEIVED.

Symptomatology and Diagnosis of Disorders of Respiration and Circulation. By Prof. Edmund von Neusser, M.D., Professor of the Second Medical Clinic, Vienna. Authorised English Translation by Andrew MacFarlane, M.D., Professor of Medical Jurisprudence and Physical Diagnosis, Albany Medical College. Part I. Dyspnea and Cyanosis. 12mo, pp. 203. New York: E. B. Treat & Co. (Cloth, $1.50.)

---

The Commoner Diseases of the Eye. By Casey A. Wood, M.D., Professor of Ophthalmology, Northwestern University, Chicago, and Thomas A. Woodruff, M.D., Ophthalmic Surgeon, St. Luke's Hospital, Chicago. 12mo, pp. 598. Illustrated. Third edition. Chicago: W. T. Keener & Co. (Cloth, $2.50.)

---

Syphilis in the Army. By Major H. C. French, Royal Army Medical Corps. Small 8 vo, pp. 134. London: John Bale, Sons & Danielsson. (Cloth, $1.50 net.)

---

Transactions of the American Otological Society. Fortieth annual meeting held at the Hotel Arlington, Washington, D. C., May 7 and 8, 1907. Vol. X, Part III. F. L. Jack, M.D., Secretary.

---

Blood Stains. Their Detection and the Determination of their Source. By Major W. D. Sutherland, M.D., of His Majesty's Indian Medical Service. Small octavo, pp. 167. Illustrated. New York: William Wood & Co.

---

Transactions of the American Surgical Association. Vol. XXV. Edited by Richard H. Harte, M.D., Recorder of the Association.

---

Modern Clinical Medicine. Vol. IV. Diseases of the Nervous System. Edited by Archibald Church, M.D., Professor of Nervous and Mental Diseases and Medical Jurisprudence in the Northwestern University Medical Department, Chicago. Authorised translation from "Die Deutsche Klinik" under the general editorial supervision of Julius I. Salinger, M.D. Octavo, pp. 1226. Illustrated. New York and London: D. Appleton & Co. (Cloth, $7.00.)

---

Heart Disease and Blood Pressure. By Louis Faugeres Bishop, A.M., M.D., Clinical Professor of Heart and Circulatory Diseases, Fordham University, New York. Second edition. 12mo. pp. 120. New York: E. B. Treat & Company. ($1.00.)

A Manual of the Practice of Medicine. By A. A. Stevens, A.M., M.D., Professor of Therapeutics and Clinical Medicine in the Woman's Medical College of Pennsylvania. Eighth Edition. 12mo of 558 pages, illustrated. Philadelphia and London: W. B. Saunders Company, 1907. (Flexible Leather, $2.50 net.)

---

The Operating Room and the Patient. By Russell S. Fowler, M.D., Professor of Surgery, Brooklyn Postgraduate Medical School, Brooklyn. Second edition. Octavo, 284 pages, fully illustrated. Philadelphia and London: W. B. Saunders Company, 1907. (Cloth, $2.00 net.)

---

Principles and Practice of Modern Otology. By John F. Barnhill, M.D., Professor of Otology, Laryngology and Rhinology in Indiana University School of Medicine, and Ernest deWolfe Wales, B.S., M.D., Associate Professor of Otology, Laryngology and Rhinology in Indiana University Medical School. Octavo, pp. 575. Illustrated. W. B. Saunders Co. (Cloth, $5.50.)

---

Kirkes' Handbook of Physiology. Sixth American Edition. Revised by Charles Wilson Greene, A.M., Ph.D., Professor of Physiology and Pharmacology in the University of Missouri. Octavo, pp. 723. Illustrated. New York: William Wood & Co. (Cloth, $3.00.)

---

A Textbook of Pathology. By Francis Delafield, M.D., LL.D., and T. Mitchell Prudden, M.D., LL.D. Eighth Edition. Octavo, 1,075 pages, illustrated by thirteen full-page plates in black and chromolithography, and by six hundred and fifty line and half-tone cuts in the text, in black and various colors. Wm. Wood & Co., New York. (Extra muslin, $5.50; leather, $6.50, net prices.

---

A Handbook of Cutaneous Therapeutics. By W. A. Hardaway, A.M., M.D., Professor of Diseases of the Skin and Syphilis, and Joseph Grindon, Ph.B., M.D., Professor of Clinical Dermatology and Syphilis in Washington University, St. Louis. 12mo, 606 pages. Lea Brothers & Co., Philadelphia and New York, 1907. (Cloth, $2.75, net.)

---

A Textbook of Physiology. By Isaac Ott, A.M., M.D., Professor of Physiology in the Medico-Chirurgical College of Philadelphia. Second Edition. Illustrated with 393 Half-tone Engravings, many in colors. Royal octavo, 815 pages. F. A. Davis Company, Philadelphia. (Cloth, $3.50 net.)

---

A Textbook of the Practice of Medicine. By James M. Anders, M.D., Ph.D., LL.D., Professor of the Theory and Practice of Medicine and of Clinical Medicine, Medico-Chirurgical College, Philadelphia. Eighth Editon. Octavo of 1317 pages, fully illustrated. Philadelphia and London: W. B. Saunders Company, 1907. (Cloth, $5.50; half morocco, $7.00 net prices.)

---

A Treatise on Diseases of the Skin. For the Use of Advanced Students and Practitioners. By Henry W. Stelwagon, M.D., Ph.D., Professor of Dermatology, Jefferson Medical College, Philadelphia. Fifth Edition. Octavo of 1,150 pages, with 267 text-illustrations, and 34 full-page colored and half-tone plates. Philadelphia and London: W. B. Saunders Company, 1907. (Cloth, $6.00; half morocco, $7.50 net prices.)

The Internal Secretions and the Principles of Medicine. By Charles E. de M. Sajous, M.D., Fellow of the College of Physicians of Philadelphia. Volume II. Octavo, pp. 1099. With 25 illustrations. Philadelphia: F. A. Davis Company. 1907.

---

The Medical Directory of New York, New Jersey and Connecticut. Published by the Medical Society of the State of New York. Volume IX. 1907.

---

Transactions of the Twenty-ninth annual meeting of the American Laryngological Association held at Washington, D. C., May 7, 8, and 9, 1907. J. E. Newcomb, M.D., Secretary.

Hospital Training School Methods and the Head Nurse. By

---

Charlotte A. Aitkens, late Director of Sibley Memorial Hospital, Washington, D. C. 12 mo. pp. 267. Philadelphia and London: W. B. Saunders Co. (Cloth, $1.50).

---

Transactions of the Section on Obstetrics and Diseases of Women of the American Medical Association at the fifty-eighth annual session held at Atlantic City, N. J., June 4-7, 1907. W. P. Manton, M.D., Secretary.

---

Surgical Applied Anatomy. By Sir Frederick Treves, F.R.C.S., Sergeant-Surgeon to H.M. the King, Late Lecturer on Anatomy at the London Hospital. New (5th) edition, thoroughly revised. Pocket size 12mo, 640 pages, 107 illustrations, of which 41 are in colors. Lea Brothers & Co., Philadelphia and New York. 1907. (Cloth, red edges, $2.25, net).

---

A Textbook of Practical Gynecology. For practitioner and students. By D. Tod Gilliam, M.D., Emeritus Professor of Gynecology in Starling-Ohio Medical College; Gynecologist to St. Anthony and St. Francis Hospitals. Second Revised Edition. Illustrated with 350 engravings, a colored frontispiece, and 13 full-page half-tone plates. 642 royal octavo pages. Sold only by subscription. F. A. Davis Company, Philadelphia. (Extra, cloth, $4.50, net; half-morocco, gilt-top, $6.00. net).

---

The Treatment of Fractures: With notes upon a few common dislocations. By Chas. L. Scudder, M.D., surgeon to the Massachusetts General Hospital. Sixth edition, revised and enlarged. Octavo volume of 635 pages, with 854 original illustrations. Philadelphia and London: W. B. Saunders Company, 1907. (Polished buckram, $5.50; half -morocco, $7.00 net prices.)

